# How co-production helped to shape Well Parent Japan: A culturally appropriate parenting intervention for mothers of children with ADHD

**DOI:** 10.1192/j.eurpsy.2023.76

**Published:** 2023-07-19

**Authors:** S. Shimabukuro

**Affiliations:** Institute of science and technology, okinawa university, okinawa, Japan

## Abstract

**Abstract:**

This presentation will talk about co-production findings from three completed studies to develop and evaluate a culturally appropriate Japanese version of the New Forest Parent Training Programme (NFPP), named Well Parent Japan (WPJ). Dr Shimabukuro will also present an on-going prospective study aiming to provide support for Japanese mothers and teachers of children with ADHD in a school setting. Dr. Shimabukuro will share the experiences worked with different stakeholders at the different stage of the study and highlight the benefits of co-production in the research and how WPJ was shaped. This presentation will also discuss difficulties and challenges when evidence-based parent training intervention is transferred from research to practice.

**Disclosure of Interest:**

None Declared

